# Behavioral Components and Context of Antimicrobial Prescription in a Tertiary Hospital in Portugal

**DOI:** 10.3390/antibiotics12061032

**Published:** 2023-06-08

**Authors:** Ana Paula Muniz Almeida-Costa, José-Artur Paiva, António Jorge Santos Almeida, Elisabete Barbosa, Sofia Correia

**Affiliations:** 1 Public Health Institute of Porto, 4050-600 Porto, Portugal; anapaulamunizcosta@vision360treinamentos.com.br; 2 Intensive Care Medicine Service at Centro Hospitalar Universitário São João (CHUSJ), 4200-319 Porto, Portugal; 3 Medicine Department at Faculty of Medicine, University of Porto, 4200-319 Porto, Portugal; jorge.salmeida@chsj.min-saude.pt; 4 Internal Medicine Service at Centro Hospitalar Universitário São João (CHUSJ), 4200-319 Porto, Portugal; 5 General Surgery Service at Centro Hospitalar Universitário São João (CHUSJ), 4200-319 Porto, Portugal; laura.barbosa@chsj.min-saude.pt; 6 ISPUP-EPIUnit—Instituto de Saúde Pública da Universidade do Porto, 4050-600 Porto, Portugal; scorreia@med.up.pt

**Keywords:** behavior, antimicrobial prescription, antimicrobial stewardship, theory of planned behavior

## Abstract

Consumption of antimicrobials is an important driver of antimicrobial resistance. There is limited knowledge of the key determinants of antimicrobial prescribing behavior in hospitals. An understanding of these determinants is required for the successful design, adoption, and implementation of quality improvement interventions in Antimicrobial Stewardship Programs (ASP). This study aimed to describe the main factors that influence the doctor’s decision on antimicrobials prescribing and to identify the behaviors that drive physicians’ decision making. A structured web-based questionnaire focused on behavioral components of antimicrobial prescription was applied to the medical staff of three different departments—Internal Medicine, General Surgery, and Intensive Care Medicine—of a university hospital. All doctors agreed that inadequate use of antimicrobials increases AMR. A total of 77% of the surgeons and 100% of the internists and intensivists perceived antimicrobial prescription as a priority in the department. Full autonomy in antimicrobial prescription was preferred by internists (64%) but not by surgeons (18%) and intensivists (24%). Most physicians were keen to have ASP advice, but most did not want advice from colleagues of the same service. Almost all surgeons ask for advice when prescribing, but only 68% of the internists do it. Less than half of all physicians and only 25% of the surgeons felt free to prescribe contrary to guidelines. Most physicians, particularly in Intensive Care Medicine (94%), adopt the “wait and see” strategy when no microbiologic confirmation is available, but 27% of the surgeons start empirical therapy. In conclusion, the context of antimicrobial prescription, autonomy, and confidence in antimicrobial prescription demonstrated heterogeneity between the three departments and this should be considered when planning ASP.

## 1. Background

Antimicrobial resistance (AMR) is a serious and growing public health problem in both hospital and community-acquired infections worldwide, with a negative impact on health and economic outcomes. Several countries face high levels of antimicrobial resistance and increasing trends are expected in the coming years [1,2]. 

Inappropriate use of antimicrobials (including over-, under-, and misuse) is recognized as a key driver of AMR. The design of antimicrobial management programs should be based on the best current understanding of the relationship between antimicrobial use and resistance [3].

Worldwide, between 2000 and 2015, antibiotic consumption, expressed in DDD, increased by 65%, and the antibiotic consumption rate increased by 39%. Projections of global antibiotic consumption in 2030, assuming no policy changes, were up to 200% higher than in 2015. The global increase in antimicrobial consumption from 2000 to 2015 was driven by low- and middle-income countries, where rising consumption correlated with gross domestic product per capita growth and, although currently lower than in high-income countries, is rapidly converging to the rates of high-income countries [4]. There were an estimated 4.95 million deaths associated with bacterial AMR in 2019, including 1.27 million deaths attributable to bacterial AMR [5]. 

In Portugal, the rates of most of the bacteria/resistance combinations, such as methicillin-resistant *Staphylococcus aureus*, vancomycin-resistant *Enterococcus faecium*, carbapenem-resistant *Acinetobacter* spp., and fluoroquinolone resistant *Escherichia coli* have been decreasing in the last decade [6]. However, carbapenem-resistant *Klebsiella pneumoniae* rates have increased from 2 to 12% in the last 8 years [6], and infections due to antibiotic-resistant bacteria are estimated to be associated with 256 disability-adjusted life years (DALYs) and 10 attributable deaths per 100,000 population [7]. Despite the implementation of different policies, hospital antibiotic consumption has not significantly improved [6], and adequate use of antibiotics is a priority target of the national priority program to combat AMR [8].

Antimicrobial stewardship programs have been implemented worldwide, particularly in the hospital setting, aiming at the promotion of more prudent use of antibiotics. They are mandatory in all health units in Portugal. Several programs have been based on audit and feedback, educational strategies, and/or formulary restriction [9]. However, contextual factors that may influence antimicrobial prescription are often disregarded [10]. In their daily practice, physicians need to balance the possible benefits and the possible individual and collective adverse events associated with antibiotic prescription and such decision making is often difficult [11]. In this context, factors such as lack of knowledge, fear of an adverse outcome, especially in more complex patients, hierarchical relations, team organization, and normative beliefs seem to be relevant drivers of antibiotic prescription [11,12]. Thus, understanding what influences antimicrobial prescription using a context-specific approach is essential to guide and optimize stewardship interventions, effectively improving prescription behavior. The theory of planned behavior is one the most used models to explain and support antibiotic prescription behavior [13]. It considers that behavior intentions are the best predictors of actual behavior and that they result from attitudes towards behaviors, subjective norms (social norms), and perceived behavioral control. These intention drivers are, in turn, influenced by individual beliefs, namely, behavioral (behaviors), normative (subjective norms), and control (behavior control), and are shaped by individual and contextual conditions [13,14].

The objective of this study is to characterize behaviors in prescribing antimicrobial drugs to hospitalized patients at a public university tertiary hospital in northern Portugal, closely examining attitudes, subjective norms, perceived behavioral control, and physicians’ intention to prescribe.

## 2. Results

The overall response rate was 61%, namely, 44% in General Surgery (*n* = 22), 60% in Internal Medicine (*n* = 39), and 82% in Intensive Care Medicine (*n* = 33).

The participants were stratified into specialists (less than 5 years as specialist); graduated specialists (between 5 to 8 years as specialist); and senior specialists (more than 8 years as specialist).

Almost 60% of the sample was under 45 years old, ranging from 50% in General Surgery to 66% in Intensive Care Medicine. Around half of participants were females, and most were specialists or graduated specialists (64%). Senior specialists represented 11% of respondents. Almost all doctors worked full-time at CHUSJ, and most had more than 10 years of experience (65%) (Table 1).

**Figure 1:**
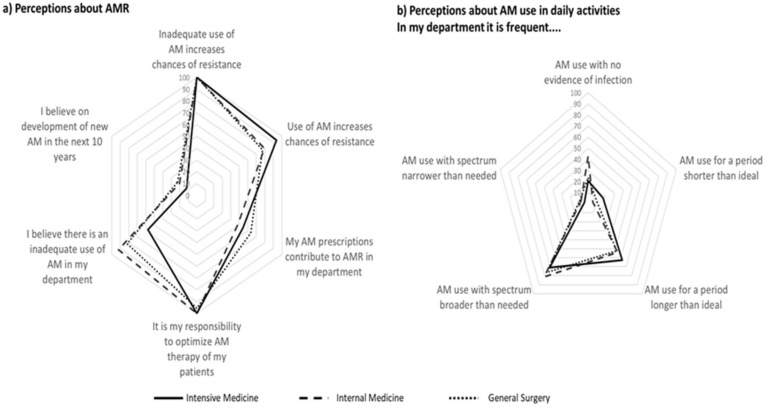
Proportion of participants that agree or totally agree with statements regarding perceptions on antimicrobial resistance (**a**) and use of antimicrobials in daily activities (**b**).

**Figure 2:**
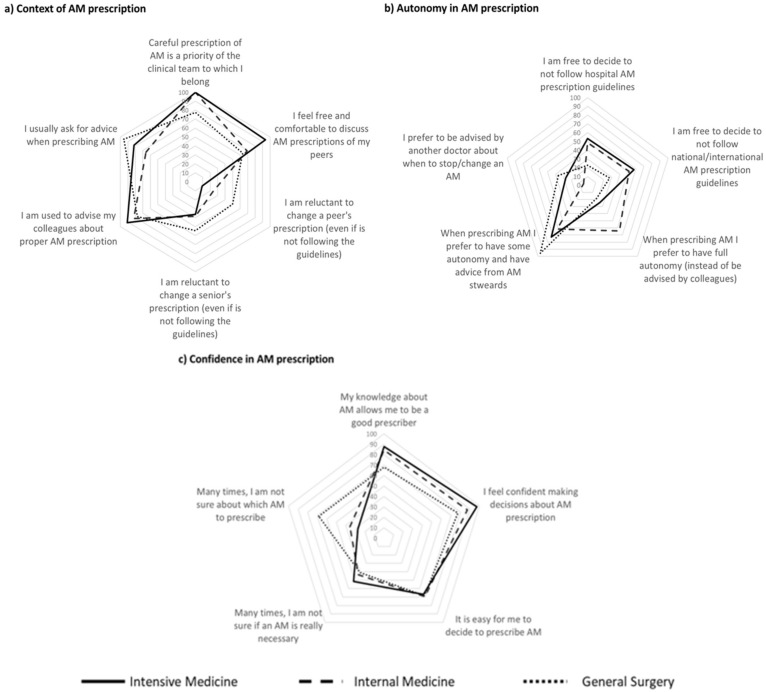
Contextual and individual characteristics influencing antimicrobial (AM) prescription decision in the three different Services assessed, in terms of (**a**) context of AM prescription; (**b**) autonomy in AM prescription and (**c**) confidence in AM prescription.

Figure 1 and Figure 2 show the proportion of physicians that agreed or totally agreed with different concepts of antimicrobial use and of AMR. Results are presented as proportions for each department. Detailed results are available in Supplementary Table S1.

All doctors agreed that inadequate use of antimicrobials increases AMR, while for 84%, overall use of antimicrobials increases AMR (almost 95% in Intensive Medicine and 77% in General Surgery). A smaller proportion reported that his/her own prescriptions contribute to AMR—from 49% in Internal Medicine to 64% in General Surgery. 

Less than 60% of the intensivists, 82% of the surgeons, and 92% of the internists agreed that inadequate antimicrobial prescription occurs in their department (Figure 1a). Around half of physicians reported departments’ use of antimicrobials for periods longer than ideal. Three out of four physicians from General Surgery and from Intensive Care Medicine reported frequent use of antimicrobials with spectra broader than needed (50% in Internal Medicine). The perception of antimicrobial use with no evidence of infection varied from 41% in Internal Medicine to 23% in General Surgery and 17% in Intensive Care Medicine (Figure 1b). Most physicians reported that it was easy to access local or national guidelines (Table 2). 

The context of antimicrobial prescription (Figure 2a) and autonomy (Figure 2b) and confidence (Figure 2c) in its prescription showed some heterogeneity between departments. 

A total of 77% of the surgeons and 100% of the internists and intensivists perceived antimicrobial prescription as a priority in the department. Being comfortable discussing prescription with peers and debating colleagues’ (even if seniors) prescriptions was more frequent among intensivists, followed by internists and general surgeons. Conversely, almost all general surgeons ask for advice when prescribing but fewer doctors (68%) from Internal medicine exhibited the same behavior (Figure 2a).

Less than half of all physicians and 25% of the surgeons felt free to prescribe contrary to guidelines. Full autonomy in antimicrobial prescription was preferred by internists (64%) but not by surgeons and intensivists (18% and 24%, respectively). Most physicians declared themselves to be keen to have advice from antimicrobial stewards, but most preferred not to be advised by colleagues of the same service to change or stop antibiotics (Figure 2b). Most physicians agreed that their knowledge about antimicrobials allows them to be good prescribers, although almost 1/3 of general surgeons did not agree with such a statement. Almost all intensivists feel confident making decisions about antimicrobial prescription, even though 50% are frequently not sure if the antimicrobial is really necessary. In General Surgery, there was a lower proportion of uncertainty regarding the need of an antimicrobial but indecision about which one to prescribe was more frequent than in the other services (68% vs. 27% in intensive care medicine). The highest confidence occurred in internal medicine, where around 43% of physicians were uncertain about the need to prescribe an antimicrobial and 36% were uncertain about which one to prescribe (Figure 2c).

**Figure 3:**
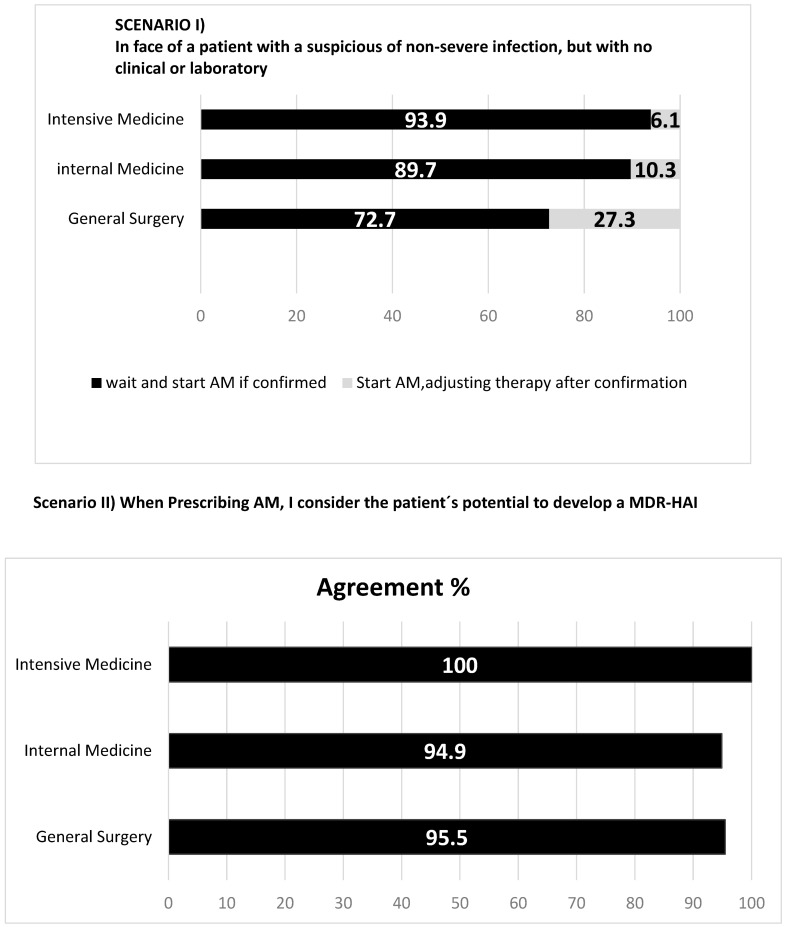

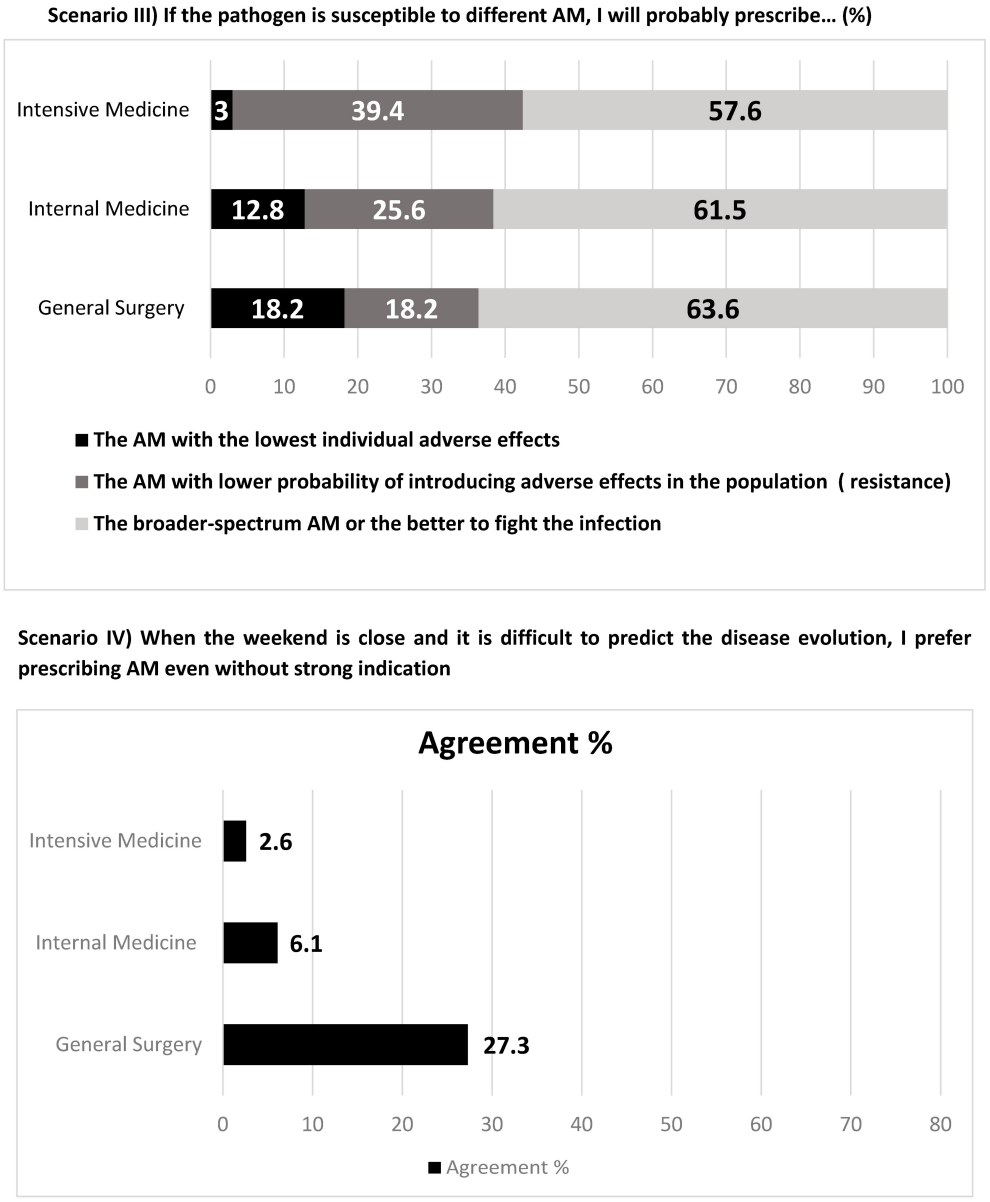
Antimicrobial (AM) prescription attitudes towards different scenarios.

Antimicrobial prescription attitudes are presented in Figure 3. Most physicians, particularly in Intensive Care Medicine (94%), adopt the “wait and see” strategy when no microbiologic confirmation is available, while 27% of general surgeons start empirical therapy in that case. When deciding which antimicrobial to prescribe, almost all consider the patients’ probability of developing resistance. When different antimicrobials are possible and appropriate, most opt to choose the one with the highest likelihood of curing the infection, almost 40% of intensivists decide for the one with lower likelihood of inducing resistance (26% in internal medicine and 18% in general surgery), and about 1/5 of general surgeons opt for the one with lowest individual adverse effects. The uncertainty of an infection and the proximity of the weekend lead 27% of surgeons to empirically prescribe an antimicrobial. On the contrary, only a residual proportion of internists and intensivists do it. When facing laboratory delays, 64% of surgeons feel they should prescribe an antimicrobial, while only 36% of intensivists and 21% of internists feel similarly. 

**Figure 4:**
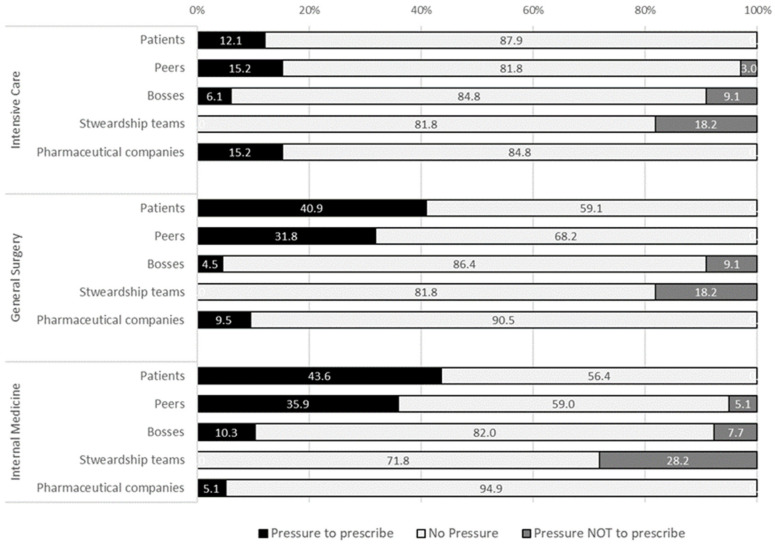
Sources of pressure towards antimicrobial prescription felt by physicians.

Regarding subjective norms, different sources of pressure towards AM prescription were observed (Figure 4). Around 40% of general surgery and internal medicine physicians felt pressure from patients to prescribe AM (12% among intensivists), and pressure not to prescribe was not felt. Peer pressure to prescribe antimicrobials was similar among internal medicine and intensive care physicians (82% and 85%), respectively. However, that number drops to 68% among surgeons.

A total of 15% of intensivists, 10% of surgeons, and 5% of internists felt pressure form pharmaceutical companies towards prescription. Finally, around 1/5 felt pressure against prescription from the local stewardship teams, mostly in the internal medicine department (28%).

For the future, most physicians intend to stop other doctors from prescribing antimicrobials without proper indication (68% in general surgery and 97% in intensive care medicine). More than 85% of general surgeons, 58% of internists, and 45% of intensivists plan to ask for local stewardship teams’ advice (Table 1).

## 3. Discussion

Our study brings to light key aspects related with the context of antimicrobial prescription. First, it reveals that departments’ leadership, prioritization of adequate antibiotic use, and team support may be related to the recognition of the problem of AMR and the ability to prescribe adequately; second, it reinforces that quality improvement, including stewardship activities, needs to be adapted to each department according to its needs, expectations, resources, and processes; third, it identifies some targets that can help the design of future effective quality improvement interventions.

Countless individual and contextual aspects can influence prescription. As observed in other settings, our study showed that professionals’ hierarchical relations, knowledge, autonomy, subjective norms, and control beliefs influence their clinical decisions [12,15,16,17,18].

This was also expressed in the pressure felt from peers towards antibiotic prescription that was lower in intensive care medicine, a department where physicians suggested less hierarchical structure. Moreover, the craving and intention to request advice differed between departments, which may limit the buy-in and the effectiveness of external stewardship interventions. While general surgeons seem to be willing to have external support, internal medicine and intensive care medicine physicians are less likely to request advice. This may be related with cultural boundaries across specialties [19], the perception of a deeper knowledge on antibiotics prescription (slightly higher in both departments when compared with general surgery), the preference for full autonomy (as particularly expressed in internal medicine), or a culture of internal discussion without the need of external peers (as observed in intensive care medicine). 

Such organizational idiosyncrasies must be specifically addressed when designing quality improvement interventions, in line with what was observed in other hospitals; prescribing etiquette and clinical leadership should be understood and taken into account before planning and implementing the intervention [12,16,20]. Understanding is a prerequisite for change; therefore, it is a necessary step before any antimicrobial stewardship interventions [21,22].

This study also demonstrates that the pressure felt by doctors can be an important factor, which brings vulnerability to actions. Pressure comes from different factors and from different directions. The pressure felt from patients (although not so strongly felt in intensive medicine, as expected because of the patients’ clinical condition) towards prescription is an interesting result that suggests the need for interventions focusing on physicians’ communication skills and patients’ engagement and literacy. It is recognized that shared clinical decision making improves the adequacy of antibiotic prescription [23]. Although this study did not evaluate the relation between doctors and pharmaceuticals’ representatives, the drug industry was still be considered a source of pressure—albeit in a lesser extent—as it has been observed in other settings [24,25].

We observed full awareness of the impact of inadequate use in antimicrobial resistance but the proportion of physicians that agreed on the influence of overall antibiotic use was lower, suggesting that the drivers of antimicrobial resistance are not fully recognized. Additionally, the perception of their own contribution to the departments’ resistance patterns was even lower in all departments. This may reflect a gap between theoretical and real risks and ascription of responsibility to others [12,15,26]. More than formal training, periodic reports on setting-specific resistance maps and feedback on physician’s prescription may be useful to decrease such a gap [12,15].

Stewardship should focus on excessively long and excessively broad-spectrum antimicrobial therapy. Although not so frequent, the use of antibiotics with no evidence of infection was also perceived as a reality, particularly in Internal Medicine. In fact, almost half of physicians are often not sure if an antibiotic is needed. These aspects may be addressed with interventions to improve the accuracy of the diagnosis, not only with the development and good use of tests but also with training on, and behavioral interventions in, dealing and tolerating the risk of a watchful waiting strategy [12]. Antimicrobial stewardship teams may provide the support and comfort for this strategy. In some departments, namely, in General Surgery, training and advice may be useful to promote the correct antibiotic to be used. In this department, physicians seem to crave support to improve their knowledge and confidence in antibiotic prescription.

Our study has some limitations. The questionnaire was developed after an extensive review of the literature and included questions designed to reflect constructs of the theory of planned behavior. However, although we tested the questionnaire in a small sample of physicians, we did not perform a formal statistical evaluation to ensure its validity and reliability. We opted to focus on only three different services of the hospital; therefore, the sample may not represent the global medical population of the hospital. However, these were the largest medical, surgical, and critical care services of the hospital, and the strategy involving heads of department resulted in a high response rate, which would not be feasible if we opted to deliver the questionnaire to all hospital physicians. Additionally, our results suggest that hospital-based approaches may lack departments’ specificities and may lead to non-effective interventions. It is also possible that physicians with some interest or knowledge in the topic tended to respond more than less interested or less informed physicians. We tried to minimize this by ensuring response anonymity with an online self-reported questionnaire. Finally, the questionnaire was opened between May and June 2020, after the first wave of COVID-19 pandemic. In that first wave, there was an increase in antimicrobial consumption at the hospital, mainly to deal with bacterial super-infections [27]. This timing may have impacted on physicians’ answers.

## 4. Participants and Methods

A cross-sectional web-survey was conducted at Centro Hospitalar Universitário São João (CHUSJ). CHUSJ is a public university and tertiary hospital with 1083 beds, and around 40,000 hospitalizations per year. The hospital has antibiotic therapy guidelines elaborated by the hospital unit for the prevention of infection and antimicrobial resistance (UPCIRA) which promotes antimicrobial stewardship activities in several clinical services.

To represent medical, surgical, and critical care areas, the three largest services of each type were selected: General Surgery (*n* = 50 medical doctors), Internal Medicine (*n* = 65 medical doctors), and Intensive Care Medicine (*n* = 40 medical doctors).

The project was presented to the heads of services together with a link to the online questionnaire (developed in Google Forms). The questionnaire was sent to the physicians by the head of service, so researchers did not have direct contact with potential participants and had no access to their contacts. The questionnaire was written in Portuguese and the form was opened for 5 weeks (between 24 May and 30 June 2020). One reminder was sent by the head of each department at the end of the fourth week (except in General Surgery). 

Participation was voluntary and anonymous. Participants were informed about the purpose, methods, and intended uses of the research and what their participation in the research entailed. Before starting the questionnaire, participants registered their acceptance in the online form. The CHUSJ ethical committee approved the study (number 55/2020).

The survey consisted of a structured questionnaire with statements that could reflect physician’s opinions. It was based in the available literature on similar surveys [15,16,17,18,19,28,29,30,31,32,33] that focused on behavioral components of antimicrobial prescription and were adapted to the Portuguese context by the research team. The theory underlying the formulation of most of the questions was based on the “Theory of Planned Behavior” [13,14], one of the theories in behavioral sciences thar describe individual behavioral patterns. Most questions were designed using a 4-point Likert Scale (strongly disagree, disagree, agree, strongly agree), and the remaining were dichotomous or multiple-choice (Supplementary Materials Table S1—English version). It was piloted in a small sample of physicians to correct for ambiguous or misunderstood questions and assess if all the relevant issues were included. 

Globally, six main sections were included in the survey:

(1) Demographic and general information: age, sex, level of training, time of experience (years), and doctor’s career position;
(2) Perceptions about local and global AMR and if physicians’ practices influence AMR;
(3) Perceived control behaviors that address how easy a prescriber feels in making a decision on antimicrobial prescription; these include self-efficiency and self-confidence and the capacity to prescribe in good practice and with a sense of control over the situation [12];
(4) Subjective norms, aimed to identify normative influences that drive doctors’ behaviors: position under different types of pressure (from peers, patients or industry, patient clinical condition, etc.) to prescribe antimicrobials;
(5) Habits and perceived knowledge regarding prescribing behavior, autonomy to change other prescribers’ decisions, attitudes towards antimicrobial prescribing;
(6) Intentions: the degree to which a prescriber is willing to change antimicrobial prescriptions and to ask for support of the hospital antimicrobial stewardship team.

Data were collected in Google Forms and stored in Microsoft Excel^®^. Data were exported to IBM SPSS Statistics 25 for analysis. The frequency of each point class of the Likert scale was calculated and then collapsed into two categories: strongly agree or agree, and strongly disagree or disagree. All variables were described using absolute (*n*) and relative (%) frequencies, presented for the overall sample and stratified by department. 

Due to the sample characteristics, age groups were collapsed into three categories—from 25–34 years, 35–54 years, and above 55 years old—and CHUSJ working years into up to 2, 3–4, and 5–10 years; and ≥10 years of experience, namely, 10–30 years and ≥30 years of experience.

## 5. Conclusions

In summary, this study demonstrates the relevance of behavioral, normative, and control aspects, together with individual and contextual conditions, on doctors’ decision-making when prescribing antimicrobials. There is not a superior strategy or a magic bullet that fits all settings, but the sum of different efforts and approaches can lead to the best outcome concerning the quality of antimicrobial prescription.

On top of the organizational and contextual department characteristics that should be taken into account in tailoring stewardship interventions towards behavioral changes, specific areas for improvement were identified regarding knowledge, the need for support, type and duration of the antimicrobials, and risk management to deal with uncertainty.

## Figures and Tables

**Table 1:** Sociodemographic data.

	Overall		Internal Medicine		General Surgery		Intensive Medicine	
	*n* = 94		*n* = 39		*n* = 22		*n* = 33	
Female	48	(51.1)	23	(59.0)	8	(36.4)	17	(51.5)
Age (years)								
25–34	32	(34.0)	16	(41.0)	8	(36.4)	8	(24.2)
35–44	23	(24.5)	6	(15.4)	3	(13.6)	14	(42.4)
45–54	14	(14.9)	5	(12.8)	4	(18.2)	5	(15.2)
55–64	22	(23.4)	10	(25.6)	6	(27.3)	6	(18.2)
≥65	3	(3.2)	2	(5.1)	1	(4.5)	0	(0.0)
Full time schedule at CHUSJ	92	(97.9)	37	(94.9)	22	100.0)	33	(100.0)
Professional category								
Resident	24	(25.5)	11	(28.2)	8	(36.4)	5	(15.2)
Specialist	27	(28.7)	10	(25.6)	2	(9.1)	15	(45.5)
Graduated Specialist	33	(35.1)	12	(30.8)	11	(50.0)	10	(30.3)
Senior Specialist	10	(10.6)	6	(15.4)	1	(4.5)	3	(9.1)
Professional experience (years)								
<10	33	(35.1)	17	(43.6)	9	(40.9)	7	(21.2)
10 to 20	24	(25.5)	5	(12.8)	1	(4.5)	18	(54.5)
21–30	15	(16.0)	7	(17.9)	6	(27.3)	2	(6.1)
>30	22	(23.4)	10	(25.6)	6	(27.3)	6	(18.2)
Working duration at CHUSJ (years)								
<1	10	(10.6)	3	(7.7)	2	(9.1)	5	(15.2)
1 to 2	6	(6.4)	1	(2.6)	2	(9.1)	3	(9.1)
3 to 4	12	(12.8)	6	(15.4)	1	(4.5)	5	(15.2)
5 to 10	18	(19.1)	9	(23.1)	5	(22.7)	4	(12.1)
>10	48	(51.1)	20	(51.3)	12	(54.5)	16	(48.5)

CHUSJ: Centro hospitalar Universitário São João.

**Table 2:** Detailed results of the questionnaire.

	Global	General Surgery	Internal Medicine	Intensive Care Medicine
	*n* (%)	*n* (%)	*n* (%)	*n* (%)
**8. My AM prescriptions contribute to AMR in my service**				
Tot. Agree	18 (19.1)	3 (13.6)	8 (20.5)	7(21.2)
Agree	33 (35.1)	11 (50.0)	11 (28.2)	11(33.3)
Disagree	37 (39.4)	6 (27.3)	20 (51.3)	11(33.3)
Tot. Disagree	6 (6.4)	2 (9.1)	0 (0.0)	4 (12.1)
**9. Inadequate use of AM increases chances of resistance to these drugs**				
Tot. Agree	87 (92.6)	20 (90.9)	35 (89.7)	32 (97.0)
Agree	7 (7.4)	2 (9.1)	4 (10.3)	1 (3.0)
Disagree	0 (0.0)	0 (0.0)	0 (0.0)	0 (0.0)
Tot. Disagree	0 (0.0)	0 (0.0)	0 (0.0)	0 (0.0)
**10. The prescription of AM increases the resistance to these drugs**				
Tot. Agree	48 (51.1)	9 (40.9)	20 (51.3)	19 (59.4)
Agree	30 (31.9)	8 (36.4)	11 (28.2)	11 (34.4)
Disagree	13 (13.8)	5 (22.7)	6 (15.4)	2 (6.3)
Tot. Disagree	2 (2.1)	0 (0.0)	2 (5.1)	0 (0.0)
**11. I believe there is an inadequate use of AM in my department**				
Tot. Agree	14 (14.9)	3 (13.6)	9 (23.1)	2 (6.1)
Agree	19 (20.2)	4 (18.2)	3 (7.7)	12 (36.4)
Disagree	59 (62.8)	15 (68.2)	27 (69.2)	17 (51.5)
Tot. Disagree	2 (2.1)	0 (0.0)	0 (0.0)	2 (6.1)
**12. In my department, antibiotic prescription often occurs in these situations:**				
Without infection evidence	26 (27.9)	5 (22.7)	16 (41)	4 (12.1)
Shorter than ideal	8 (8.5)	2 (9.1)	2 (5.1)	4 (12.1)
Longer than ideal	46 (48.9)	11 (50.0)	20 (51.3)	16 (48.5)
Wider than ideal	63 (67.0)	16 (72.7)	30 (76.9)	17 (51.5)
Narrow than ideal	6 (6.4)	2 (9.1)	1 (3.0)	3 (7.7)
**13. It is my responsibility, as health professional, to help all around me about the correct use of AM**				
Tot. Agree	70 (74.5)	15 (68.2)	28 (71.8)	27 (81.8)
Agree	24 (24.5)	7 (31.8)	11 (28.2)	6 (18.2)
Disagree	0 (0.0)	0 (0.0)	0 (0.0)	0 (0.0)
Tot. Disagree	0 (0.0)	0 (0.0)	0 (0.0)	0 (0.0)
**14. It is my responsibility to optimize the antibiotic therapy of my patients**				
Tot. Agree	79 (84.0)	15 (68.2)	34 (87.2)	30 (90.9)
Agree	14 (14.9)	6 (27.3)	5 (12.8)	3 (9.1)
Disagree	1 (1.1)	1 (4.5)	0 (0.0)	0 (0.0)
Tot. Disagree	0 (0.0)	0 (0.0)	0 (0.0)	0 (0.0)
**15. I believe on development of new antibiotics in the next 10 years**				
Tot. Agree	0 (0.0)	0 (0.0)	0 (0.0)	0 (0.0)
Agree	17 (18.1)	5 (22.7)	8 (20.5)	4 (12.1)
Disagree	57 (60.6)	14 (63.6)	21 (53.8)	22 (66.7)
Tot. Disagree	20 (21.3)	3 (13.6)	10 (25.6)	7 (21.2)
**16. In general, the AM guidelines are not adequate for those to whom I prescribe AM**				
Tot. Agree	0 (0.0)	0 (0.0)	0 (0.0)	0 (0.0)
Agree	16 (17.0)	2 (9.1)	8 (20.5)	6 (18.2)
Disagree	70 (74.5)	17 (17.3)	29 (74.4)	24 (72.7)
Tot. Disagree	8 (8.5)	3 (13.6)	2 (5.1)	3 (9.1)
**17. I know how to find AM guidelines in my dept./hospital**				
Tot. Agree	19 (20.2)	3 (13.6)	11 (28.2)	5 (15.2)
Agree	50 (50.3)	10 (45.5)	22 (56.4)	18 (54.5)
Disagree	23 (24.5)	9 (40.9)	6 (15.4)	8 (24.2)
Tot. Disagree	2 (2.1)	0 (0.0)	0 (0.0)	2 (6.1)
**18. I know how to find AM national guidelines**				
Tot. Agree	19 (20.2)	3 (13.6)	10 (26.3)	6 (18.2)
Agree	60 (63.8)	11 (50.0)	26 (68.4)	23 (69.7)
Disagree	13 (13.8)	8 (36.4)	2 (5.3)	3 (9.1)
Tot. Disagree	1 (1.1)	0 (0.0)	0 (0.0)	1 (3.0)
**19. I prefer to have full autonomy to prescribe AM than to be oriented by another colleague**				
Tot. Agree	8 (8.5)	0 (0.0)	5 (12.8)	3 (9.1)
Agree	29 (30.9)	4 (18.2)	20 (51.3)	5 (15.2)
Disagree	47 (50.0)	14 (63.6)	13 (33.3)	20 (60.6)
Tot. Disagree	10 (10.6)	4 (18.2)	1 (2.6)	5 (15.2)
**20. I prefer to have some orientation from the AMR Prevention Program Team when prescribing AM**				
Tot. Agree	16 (17.0)	10 (45.5)	4 (10.3)	2 (6.1)
Agree	53 (56.4)	11 (50.0)	20 (51.3)	22 (66.7)
Disagree	22 (23.4)	1 (4.5)	14 (35.9)	7 (21.2)
Tot. Disagree	3 (3.2)	0 (0.0)	1 (2.6)	2 (6.1)
**21. I am free to decide not to follow the guidelines on AM prescription of my Dept./Hospital**				
Tot. Agree	6 (6.4)	0 (0.0)	1 (2.6)	5 (15.6)
Agree	35 (37.2)	5 (22.7)	18 (46.2)	12 (37.5)
Disagree	45 (47.9)	14 (63.6)	17 (43.6)	14 (43.8)
Tot. Disagree	7 (7.4)	3 (13.6)	3 (7.7)	1 (3.1)
**22. I am free to decide not to follow the national/international guidelines on AM use**				
Tot. Agree	4 (4.3)	0 (0.0)	1 (2.6)	3 (9.1)
Agree	41 (43.6)	6 (27.3)	19 (48.7)	16 (48.5)
Disagree	38 (40.4)	11 (50.0)	17 (43.6)	10 (30.3)
Tot. Disagree	11 (11.7)	5 (22.7)	2 (5.1)	4 (12.1)
**23. I am reluctant to change AM prescription from senior colleagues (even if not concordant with recommendations)**				
Tot. Agree	8 (8.5)	3 (13.6)	4 (10.3)	1 (3.0)
Agree	31 (33.0)	9 (40.9)	11 (28.2)	11(33.3)
Disagree	44 (46.8)	7 (31.8)	19 (48.7)	18 (54.5)
Tot. Disagree	11 (11.7)	3 (13.6)	5 (12.8)	3 (9.1)
**24. I am reluctant to change AM prescription from any colleague (even if not concordant with recommendations)**				
Tot. Agree	5 (5.3)	2 (9.1)	3 (7.7)	0 (0.0)
Agree	18 (19.1)	9 (40.9)	6 (15.4)	3 (9.1)
Disagree	58 (61.7)	8 (36.4)	24 (61.5)	26 (78.8)
Tot. Disagree	13 (13.8)	3 (13.6)	6 (15.4)	4 (12.1)
**25. I used to advise other colleagues about which AM should be prescribed**				
Tot. Agree	12 (12.8)	3 (13.6)	6 (15.4)	3 (9.1)
Agree	67 (71.3)	14 (63.6)	26 (66.7)	27 (81.8)
Disagree	13 (13.8)	5 (22.7)	7 (7.9)	1 (3.0)
Tot. Disagree	2 (2.1)	0 (0.0)	0 (0.0)	2 (6.1)
**26. I am not often sure about which AM to prescribe**				
Tot. Agree	3 (3.2)	1 (4.5)	2 (5.1)	0 (0.0)
Agree	35 (37.2)	14 (63.6)	12 (30.8)	9 (27.3)
Disagree	53 (56.4)	7 (31.8)	23 (59.0)	23 (69.7)
Tot. Disagree	3 (3.2)	0 (0.0)	2 (5.1)	1 (3.0)
**27. Prudent prescription of AM is a priority for the clinical team to which I belong**				
Tot. Agree	53 (56.4)	8 (36.4)	20 (51.3)	25 (75.8)
Agree	36 (38.3)	9 (40.9)	19 (48.7)	8 (24.2)
Disagree	5 (5.3)	5 (22.7)	0 (0.0)	0 (0.0)
Tot. Disagree	0 (0.0)	0 (0.0)	0 (0.0)	0 (0.0)
**28. I feel free and comfortable to question my peers’ AM prescriptions**				
Tot. Agree	36 (38.3)	5 (22.7)	10 (25.6)	21 (63.6)
Agree	36 (38.3)	9 (40.9)	17 (43.6)	10 (30.3)
Disagree	22 (23.4)	8 (36.4)	12 (30.8)	2 (6.1)
Tot. Disagree	0 (0.0)	0 (0.0)	0 (0.0)	0 (0.0)
**29. I used to ask for advice when prescribing antimicrobials**				
Tot. Agree	20 (21.3)	6 (28.6)	5 (13.2)	9 (27.3)
Agree	53 (56.4)	14 (66.7)	21 (55.3)	18 (54.5)
Disagree	18 (19.1)	1 (4.8)	12 (31.6)	5 (15.2)
Tot. Disagree	1 (1.1)	0 (0.0)	0 (0.0)	1 (3.0)
**30. The knowledge that I have about AM is enough to be able to prescribe adequately**				
Tot. Agree	5 (5.3)	0 (0.0)	2 (5.1)	3 (9.1)
Agree	72 (76.6)	15 (68.2)	31 (79.5)	26 (78.8)
Disagree	17 (18.1)	7 (31.8)	6 (15.4)	4 (12.1)
Tot. Disagree	0 (0.0)	0 (0.0)	0 (0.0)	0 (0.0)
**31. Frequently, I am not sure about which AM to prescribe**				
Tot. Agree	3 (3.2)	1 (4.5)	1 (2.6)	1 (3.0)
Agree	40 (42.6)	8 (36.4)	16 (41.0)	16 (48.5)
Disagree	46 (48.9)	12 (54.5)	20 (51.3)	14 (42.4)
Tot. Disagree	5 (5.3)	1 (4.5)	2 (5.1)	2 (6.1)
**32. I prefer to be advised by another doctor about when to stop/change an AM**				
Tot. Agree	0 (0.0)	0 (0.0)	0 (0.0)	0 (0.0)
Agree	19 (20.2)	8 (36.4)	9 (27.3)	2 (5.1)
Disagree	64 (68.1)	14 (63.6)	29 (74.4)	21 (63.6)
Tot. Disagree	11 (11.7)	0 (0.0)	8 (20.5)	3 (9.1)
**33. When prescribing AM, I consider the risk of a future development of an infection due to a MDR microorganism**				
Tot. Agree	39 (41.5)	7 (31.8)	13 (33.3)	19 (59.4)
Agree	51 (54.3)	14 (63.6)	24 (61.5)	13 (40.6)
Disagree	3 (3.2)	1 (4.5)	0 (0.0)	2 (5.1)
Tot. Disagree	0 (0.0)	0 (0.0)	0 (0.0)	0 (0.0)
**34. I prescribe an AM without strong indication when I am uncertain about disease evolution and the weekend is close**				
Tot. Agree	0 (0.0)	0 (0.0)	0 (0.0)	0 (0.0)
Agree	9 (9.6)	6 (27.3)	1 (2.6)	2 (6.1)
Disagree	47 (50.0)	10 (45.5)	23 (59.0)	14 (42.4)
Tot. Disagree	38 (40.4)	6 (27.3)	15 (38.5)	17 (51.5)
**Concerning the pressure about prescribing antimicrobials, I feel pressured by:**				
**35. …patients**				
NO pressure	54 (57.4)	13 (59.1)	22 (56.4)	29 (87.8)
Some pressure	37 (39.4)	9 (40.9)	17 (43.6)	4 (12.1)
Some pressure NOT to prescribe	3 (3.2)	1 (4.5)	0 (0.0)	1 (2.6)
**36. …my colleagues**				
NO pressure	65 (69.1)	15 (68.2)	23 (59.0)	27 (81.8)
Some pressure	26 (27.9)	7 (31.8)	14 (35.9)	5 (15.2)
Some pressure NOT to prescribe	3 (3.2)	0 (0.0)	2 (5.1)	1 (3.0)
**37. …my bosses**				
NO pressure	79 (84.0)	19 (86.4)	32 (82.1)	28 (84.8)
Some pressure	7 (7.4)	1 (4.5)	4 (10.3)	2 (6.1)
Some pressure NOT to prescribe	8 (8.5)	2 (9.1)	3 (7.7)	3 (9.1)
**38. …the hospital’s AMR prevention program team/antimicrobial stewardship team**				
NO pressure	73 (77.0)	18 (81.8)	28 (71.8)	27 (81.8)
Some pressure	0 (0.0)	0 (0.0)	0 (0.0)	0 (0.0)
Some pressure NOT to prescribe	21 (22.3)	4 (18.2)	11 (28.2)	6 (18.2)
**39. … pharmaceutical companies**				
NO pressure	84 (90.3)	19 (90.5)	37 (94.9)	28 (84.8)
Some pressure	9 (9.7)	2 (9.5)	2 (5.1)	5 (15.2)
Some pressure NOT to prescribe	0 (0.0)	0 (0.0)	0 (0.0)	0 (0.0)
**40. I believe that I should prescribe AM when the patient wants to come back to work**				
Tot. Agree	0 (0.0)	0 (0.0)	0 (0.0)	0 (0.0)
Agree	2 (2.1)	1 (4.5)	1 (3.0)	0 (0.0)
Disagree	32 (34.0)	8 (36.4)	12 (30.8)	12 (36.4)
Tot. Disagree	60 (63.8)	13 (59.1)	27 (69.2)	20 (60.6)
**41. I believe I should prescribe AM when the microbiology laboratory delays results**				
Tot. Agree	5 (5.3)	0 (0.0)	4 (10.3)	1 (3.0)
Agree	29 (30.9)	14 (63.6)	4 (10.3)	11(33.3)
Disagree	32 (34.0)	5 (22.7)	18 (46.2)	9 (27.3)
Tot. Disagree	28 (29.8)	3 (13.6)	13 (33.3)	12 (36.4)
**42. I think that deciding on AM prescription to patients is easy**				
Tot. Agree	1 (1.1)	0 (0.0)	1 (2.6)	0 (0.0)
Agree	52 (55.3)	14 (63.6)	20 (51.3)	18 (54.5)
Disagree	39 (41.5)	8 (36.4)	17 (43.6)	14 (42.4)
Tot. Disagree	2 (2.1)	0 (0.0)	1 (2.6)	1 (3.0)
**43. To prescribe or not to prescribe AM depends totally on me**				
Tot. Agree	6 (6.4)	3 (13.6)	3 (7.7)	0 (0.0)
Agree	29 (30.9)	5 (22.7)	19 (48.7)	5 (15.2)
Disagree	55 (58.5)	13 (59.1)	15 (38.5)	27 (81.8)
Tot. Disagree	4 (4.3)	1 (4.5)	2 (5.1)	1 (3.0)
**44. I feel confident to make a decision about AM prescribing**				
Tot. Agree	12 (12.8)	2 (9.1)	6 (15.4)	4 (12.1)
Agree	71 (75.5)	15 (68.2)	28 (71.8)	28 (84.8)
Disagree	11 (11.7)	5 (22.7)	1 (3.0)	5 (12.8)
Tot. Disagree	0 (0.0)	0 (0.0)	0 (0.0)	0 (0.0)
**45. It is easy for me to decide if I should or shouldn’t prescribe AM**				
Tot. Agree	2 (2.1)	1 (4.5)	1 (2.6)	0 (0.0)
Agree	62 (66.0)	14 (63.6)	26 (66.7)	22 (66.7)
Disagree	30 (31.9)	7 (31.8)	12 (30.8)	11(33.3)
Tot. Disagree	0 (0.0)	0 (0.0)	0 (0.0)	0 (0.0)
**46. When treating a non-severe patient with suspected infection, without clinical or laboratory confirmation**				
I await and establish antimicrobial therapy only if confirmed	82 (87.2)	16 (72.7)	35 (89.7)	31 (93.9)
I start the antimicrobial treatment, adjusting further the results	12 (12.8)	6 (27.3)	4 (10.3)	2 (6.1)
**47. If the microorganism is susceptible to more than one antimicrobial, I will probably decide on:**				
…the AM with less adverse effects to the patient	10 (10.6)	4 (18.2)	5 (12.8)	1 (3.0)
…the AM with less probability to induce adverse community effect (induce resistance)	27 (28.7)	4 (18.2)	10 (25.6)	13 (39.4)
…the one with better broad-spectrum or best possibility to work against the infection	57 (60.6)	14 (63.6)	24 (61.5)	19 (57.6)
**48. I plan to continue prescribing antimicrobials as I do it now, regardless of the support I may have**				
Tot. Agree	1 (1.1)	0 (0.0)	1 (2.6)	0 (0.0)
Agree	37 (39.4)	7 (31.8)	15 (38.5)	15 (45.5)
Disagree	50 (53.2)	12 (54.5)	22 (56.4)	16 (48.5)
Tot. Disagree	6 (6.4)	3 (13.6)	1 (2.6)	2 (6.1)
**49. I plan to reduce my antimicrobial prescriptions**				
Tot. Agree	4 (4.3)	0 (0.0)	2 (5.1)	2 (6.1)
Agree	33 (35.1)	11 (50.0)	10 (25.6)	12 (36.4)
Disagree	57 (60.6)	11 (50.0)	27 (69.2)	19 (57.6)
Tot. Disagree	0 (0.0)	0 (0.0)	0 (0.0)	0 (0.0)
**50. I plan to stop the prescription of antimicrobials made by other doctors, who do not have an adequate indication**				
Tot. Agree	19 (20.2)	2 (9.1)	8 (20.5)	9 (27.3)
Agree	60 (63.8)	13 (59.1)	24 (61.5)	23 (69.7)
Disagree	15 (16.0)	7 (31.8)	7 (17.9)	1 (3.0)
Tot. Disagree	0 (0.0)	0 (0.0)	0 (0.0)	0 (0.0)
**51. I plan to ask the antimicrobial stewardship team for advice**				
Tot. Agree	6 (6.5)	3 (13.6)	2 (5.3)	1 (3.0)
Agree	50 (53.8)	16 (72.7)	20 (52.6)	14 (42.4)
Disagree	37 (39.8)	3 (13.6)	16 (42.1)	18 (54.5)
Tot. Disagree	0 (0.0)	0 (0.0)	0 (0.0)	0 (0.0)

## Data Availability

Data will be available upon request to the research team.

## Supplementary Materials

The following supporting information can be downloaded at: https://www.mdpi.com/article/10.3390/antibiotics12061032/s1, Table S1: Survey Instrument, version in English.
