# Supplementary material for: Behavioral Components and Context of Antimicrobial Prescription in a Tertiary Hospital in Portugal
Source: Antibiotics (Basel). 2023 Jun 8;12(6):1032. doi: 10.3390/antibiotics12061032 (PMC10295435; doi:10.3390/antibiotics12061032)
Supplement: Supplementary file 1 [file antibiotics-12-01032-s001.zip › antibiotics-2400696-supplementary.pdf]

antibiotics

MDPI

Supplementary Materials

Table S1. Survey Instrument, version in English.

1.Age
2.Gender
3.Including internship time, how many years of experience do you have?
4.What is your current Specialty (attending only)?
5.What is your working hour at CHUSJ?
6.How long have you worked at CHUSJ?
7.Which department do you belong to?
8.My AM prescriptions contribute to AMR in my service
9.Inadequate use of AM increase chances of Resistance of these drugs
10.The prescription of AM increases the resistance to these drugs
11.I believe there is an inadequate use of AM in my department
12.In my daily activities (mine or from my department) is used to prescribe AM in the follow situations:
Without infection evidence
Shorter than ideal
Longer than ideal
With wider-spectrum antimicrobials than ideal
With narrow-spectrum antimicrobials than ideal
13. It's my responsibility, as health professional, to help all around me concerned to correct use of AM
14. It's my responsibility to optimize the antibiotic therapy of my patients
15. I believe on development of new antibiotics in the next 10 years
16. In general, the AM guidelines are not adequate to those whom I prescribe AM
17. I know how to find AM guidelines in my dept./hospital
18. I know how to find AM national guidelines
19. I prefer to have full autonomy to prescribe AM than to be oriented by another colleague
20. I prefer to have just some autonomy and have some orientation from the Program of Control of AMR ("UPCIRA") when prescribing AM than do it by
21. I am free to decide not to follow the guidelines of AM prescribing of my Dept./Hospital
22. I am free to decide not to follow the national/international guidelines of AM prescribing
23. I am reluctant in change AM prescription from senior colleagues (even if not adequate to the recommendations)
24. I am reluctant in change AM prescription from any colleague (even if not adequate to the recommendations)
25. I used to advice other colleagues about which AM should them to prescribe
26. I am not often sure about which AM to prescribe
27. Prudent prescription of AM is a priority for the clinical team to which I belong
28. I feel free and comfortable to question my peers' AM prescriptions
29. I used to ask for advice when prescribing antimicrobials
30. The knowledge that I have about AM is enough to me to make a good prescription
31. Frequently, I am not sure about which AM to prescribe
32. I prefer to be advised by another doctor about when to stop/change an AM
33. When prescribing AM, I consider the potential that the patient has of developing a health care-associated infection with a multi-resistant microorganism
34. I prescribe an AM even without strong indication when the end of the week is approaching and it is still difficult to predict the course of the disease
According to the pressure about prescribing antimicrobials I feel pressured by:
35. Patients
36.My colleagues
37.My superiors
38.PAPA/UPCIRA
39.From Pharmaceutical companies
40. I believe that I should prescribe AM when the patient wants to come back to work
41. I believe that I should prescribe AM when there is a delay from the clinical Laboratory to deliver the antibiogram results

42. Making a decision about AM prescription to patients is somehow easy
43. To prescribe or not AM, depend totally on me
44. I feel confident to make a decision about AM prescribing
45. It's easy to me to decide if I should or shouldn't to prescribe AM
46. When treating a patient with suspected infection, without clinical or laboratory confirmation, and without severity
...I await and establish antimicrobial therapy only if confirmed
... I start the antimicrobial treatment, adjusting it after the results
47. When I receive the results (antibiogram) and I realize the microorganism is sensible to more than one antimicrobial, I'll probably decide for:
The AM with less adverse effects to the patient
The AM with less probability to induce adverse community effect (induce resistance)
I start the antimicrobial treatment, adjusting further the results
48. I plan to continue prescribing antimicrobials to patients the way I always have, regardless of having more or less support
49. I plan to reduce my antimicrobial prescriptions
50. I plan to stop the prescription of antimicrobials, made by other doctors, who do not have an adequate indication
51. I plan to ask to Control of infections Department "PAPA/UPCIRA" advices
